# Cancer Incidence and Outcomes After Kidney Transplantation in Balkan Nephropathy Patients

**DOI:** 10.3390/jcm15166226

**Published:** 2026-08-12

**Authors:** Matko Prtorić, Armin Atić, Bojan Jelaković, Željko Kaštelan, Nikolina Bašić-Jukić

**Affiliations:** 1 School of Medicine, University of Zagreb, 10000 Zagreb, Croatia; matko.prtoric35@gmail.com (M.P.);; 2 Department of Nephrology, Arterial Hypertension, Dialysis and Transplantation, University Hospital Center Zagreb, 10000 Zagreb, Croatia; 3 Urology Clinic, University Hospital Center Zagreb, 10000 Zagreb, Croatia

**Keywords:** aristolochic acid, Balkan nephropathy, urothelial carcinoma, kidney transplantation, long-term survival

## Abstract

**Background**: Balkan nephropathy (BEN), caused by chronic dietary exposure to aristolochic acid (AA), is a tubulointerstitial kidney disease strongly associated with upper-tract urothelial carcinoma (UTUC). Patient and graft survival, the cumulative incidence and timing of post-transplant UTUC, and the burden of non-urothelial malignancy after kidney transplantation remain poorly defined. **Methods**: We retrospectively analyzed all patients with BEN as the primary cause of end-stage kidney disease transplanted at University Hospital Center Zagreb between October 1973 and December 2023. Outcomes included patient and graft survival, the incidence and timing of post-transplant UTUC, and burden of non-urothelial malignancies. **Results**: Of 2282 kidney transplants performed in the study period, 44 (2%) were in patients with BEN. The median age at transplantation was 56 years, and the median dialysis vintage was 3.7 years. After 10 years (median) of follow up, among 34 patients with adequate follow-up, 16 (47%) developed a de novo malignancy: eight (24%) UTUC, one renal cell carcinoma, four other solid tumors, and three hematological. UTUC was diagnosed between 6 months and 15 years post-transplant (median 7 years); five of eight patients died within one year of diagnosis. Five- and ten-year patient survival rates were 82% and 54.5%. **Conclusions**: Kidney transplantation in patients with BEN carries a lifelong cancer risk dominated by UTUC. Prophylactic bilateral nephroureterectomy should be the default management strategy when feasible, with lifelong surveillance extending beyond the urothelium.

## 1. Introduction

Balkan nephropathy (BEN) is a chronic kidney disorder that has affected rural communities along the Danube River and its tributaries for many decades. It is characterized by progressive tubulointerstitial damage and an increased risk of UTUC, making it distinct among other causes of end-stage kidney disease (ESKD) [1]. Although its incidence has declined in recent years due to improvements in agricultural and milling technology followed by socioeconomic betterment and preventive efforts, BEN remains a paradigmatic example of an environmentally induced chronic kidney disease—that is, a well-characterized human model of tubulointerstitial injury caused by a defined environmental nephrotoxin—and of its long-term carcinogenic consequences [2].

The causative agent is aristolochic acid (AA), a phytotoxin from *Aristolochia clematitis* and one of the most potent naturally occurring carcinogens. Chronic exposure to this environmental factor culminates in the pathological findings of BEN, characterized by tubular atrophy and interstitial fibrosis that are more severe in the outer cortex than in the inner cortex, and, with disease progression, glomerular and vascular lesions. Kidneys are notably small and contracted at the time of ESKD [3,4]. AA forms covalent DNA adducts at deoxyadenosine residues, producing a near-pathognomonic A→T transversion mutational pattern designated SBS22, which has been identified in upper-tract urothelial cancer (UTUC), bladder cancer, renal cell carcinoma, hepatocellular carcinoma and intrahepatic cholangiocarcinoma [5,6,7]. Today, BEN is considered an environmental form of aristolochic acid nephropathy (AAN).

BEN can only be definitively confirmed by detecting aristolactam–DNA adducts and the characteristic A:T→T:A transversion signature; however, these tests are largely unavailable. In epidemiological and clinical practice, BEN has historically been diagnosed using combinations of epidemiological, clinical, and laboratory findings, and diagnostic and classification criteria have been developed by an expert group and adopted internationally [8]. However, these criteria are not consistently applied in routine practice, which may lead to an overestimation of BEN in endemic areas and underdiagnosis outside recognized endemic villages (so-called sporadic BEN). The most important consequence of a misdiagnosis concerns the decision whether to perform prophylactic bilateral nephroureterectomy before kidney transplantation.

Kidney transplantation is the treatment of choice for patients with BEN and ESKD, with several specific clinical points: BEN patients should be considered for bilateral nephroureterectomy before transplantation; those who decline the procedure require close monitoring for post-transplant UTUC; and a potential reduction in immunosuppression intensity, along with conversion to mTOR inhibitors, should be evaluated by the clinical team [8].

Previous reports on kidney-transplant recipients with BEN have included small cohorts and often limited follow-up, leaving uncertainty about graft survival, UTUC incidence, and post-transplant outcomes [9,10].

The primary objective of this study was to determine the cumulative incidence, timing, and outcomes of post-transplant UTUC in kidney-transplant recipients who were diagnosed as BEN patients with up to 10 years of follow-up. Secondary objectives were to describe overall patient and graft survival, as well as the spectrum of non-urothelial malignancies, and to compare baseline characteristics between patients who did and did not develop UTUC.

## 2. Materials and Methods

This is a retrospective observational study of medical records of kidney transplant recipients who were diagnosed as BEN and were transplanted at University Hospital Center Zagreb from October 1973 to December 2023. The aim of this study was to evaluate long-term post-transplantation outcomes, including graft and patient survival and the prevalence of urothelial and other malignant diseases. The data collected included demographic information, clinical examination results, laboratory and radiological test results, and surgical and other medical notes. For patients diagnosed with UTUC, data on occurrence, stage, treatment, and outcomes were collected. Data were collected by two researchers who compiled a single database using electronic health medical records (BIS, IN2 grupa, Zagreb, Croatia) as well as paper medical records stored in the hospital archive for patients transplanted prior to 2011 (the year of implementation of the current electronic system). The data were further independently validated by two senior researchers. The data used were collected during routine clinical work and not for the purpose of this study.

All patients who underwent transplantation at UHC Zagreb from October 1973 to December 2023, in whom BEN was the primary diagnosis, were included in the study. For baseline cohort description we included all 40 evaluable patients. The cancer-incidence analyses were restricted to the 34 patients with adequate post-transplant follow-up data. For survival analyses, patients with partial follow-up data were censored at the last documented contact.

BEN diagnosis was based on the diagnosis recorded by the treating nephrologist at the time of transplantation; cases were not reclassified retrospectively. To reduce misclassification, two senior researchers independently reviewed each diagnosis against the source records, and cases that could not be substantiated by residential, clinical, or histopathological evidence were excluded. De novo malignancies were identified through routine post-transplant surveillance at UHC Zagreb and a review of hospital pathology records, and all malignancies were classified according to the corresponding histopathological report. Adequate follow-up was defined a priori as the availability of documented cancer-screening or diagnostic data, including urine cytology, upper-tract imaging, and/or histopathology, after transplantation or until an earlier event, such as malignancy, graft loss, or death. Incomplete ascertainment among patients who transferred care is acknowledged as a study limitation. Missing data are reported in Supplementary Table S1.

Statistical analysis was performed using IBM SPSS software, version 26.0 (IBM Corp. Released 2019. IBM SPSS Statistics for Windows, Version 26.0. Armonk, NY, USA: IBM Corp). Continuous variables are summarized as the median (with mean and standard deviation where informative) and compared between two groups with the Mann–Whitney U test; normally distributed variables, where normality was supported by the Shapiro–Wilk test, were compared with Student’s *t*-test. Categorical variables were compared with the chi-square test or Fisher’s exact test. Given the small number of events, logistic regression and survival analyses (Kaplan–Meier estimates with the log-rank test) were regarded as exploratory and hypothesis-generating; candidate covariates were limited to clinically pre-specified variables (age, sex, dialysis vintage, and viral reactivation) to avoid over-fitting. The limited sample size and event count confer a substantial risk of type II error, and non-significant comparisons should not be interpreted as evidence of an absence of an effect. All values were deemed statistically significant at a two-tailed *p* < 0.05. Figures were generated in Python (version 3.11) using the matplotlib (version 3.8) plotting library. Generative AI (Claude.ai, Anthropic) was used solely to assist in generating figure-plotting code; it was not used for data collection, data interpretation, statistical analysis, or the writing of the manuscript, and all AI-assisted output was reviewed and verified by the authors.

The Ethics Committee of University Hospital Center Zagreb approved the study (No. 8.1.-25/203-2) on 9 September 2025. The study complied with the Declaration of Helsinki and its later amendments. Because of the retrospective design, the Committee waived the requirement for written informed consent.

## 3. Results

### 3.1. Cohort Characteristics

**Figure 1:**
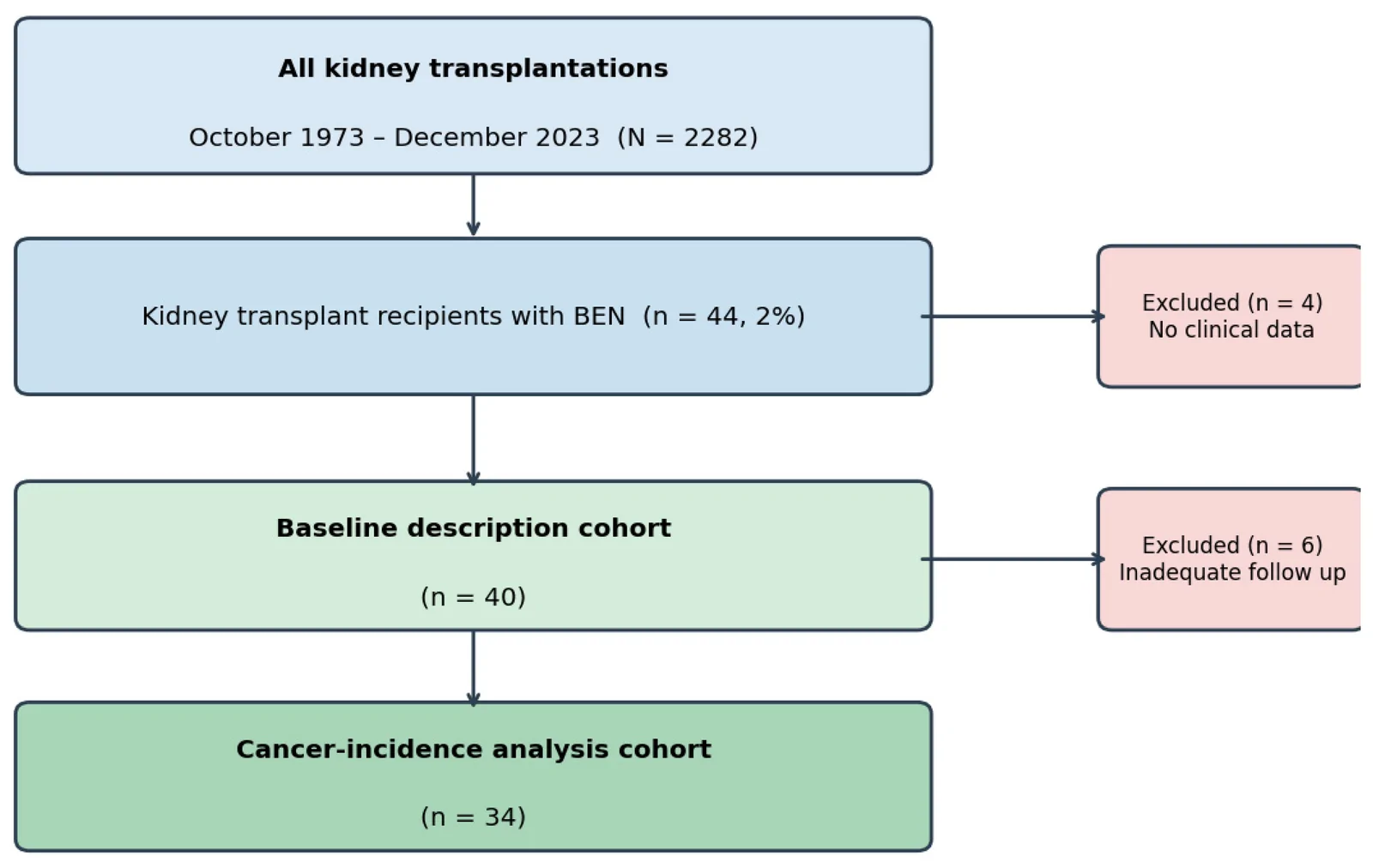
Flow diagram.

There were 2282 kidney transplantations in the studied period, with 44 (2%) of patients having BEN as the primary kidney disease (Figure 1). Nearly half of the patients were female (45%), with a median age at transplantation of 56 years (mean: 55 ± 10 years) and a median dialysis vintage of 3.7 years (mean: 4.4 ± 3 years). All the patients received their kidney from donors after brain death. In all cases this was the first transplantation event. Two patients had UTUC prior to kidney transplantation. One patient underwent nephroureterectomy several years prior to transplantation, while the other had renal carcinoma discovered incidentally during a histopathological examination of the ectomized kidney during the transplantation event. He was the only patient in whom a prophylactic bilateral nephroureterectomy was performed.

The median follow-up duration was 10.5 years (mean: 11.6; range: 0–28 years). Adequate follow-up data were available for 34 patients. All patients included in the survival analysis either reached the event or got censored at 10 years; none were lost to follow-up prior to that time point. The missing data are presented in Supplementary Table S1.

### 3.2. Upper-Tract Urothelial Cancer and Other Malignant Diseases

**Figure 2:**
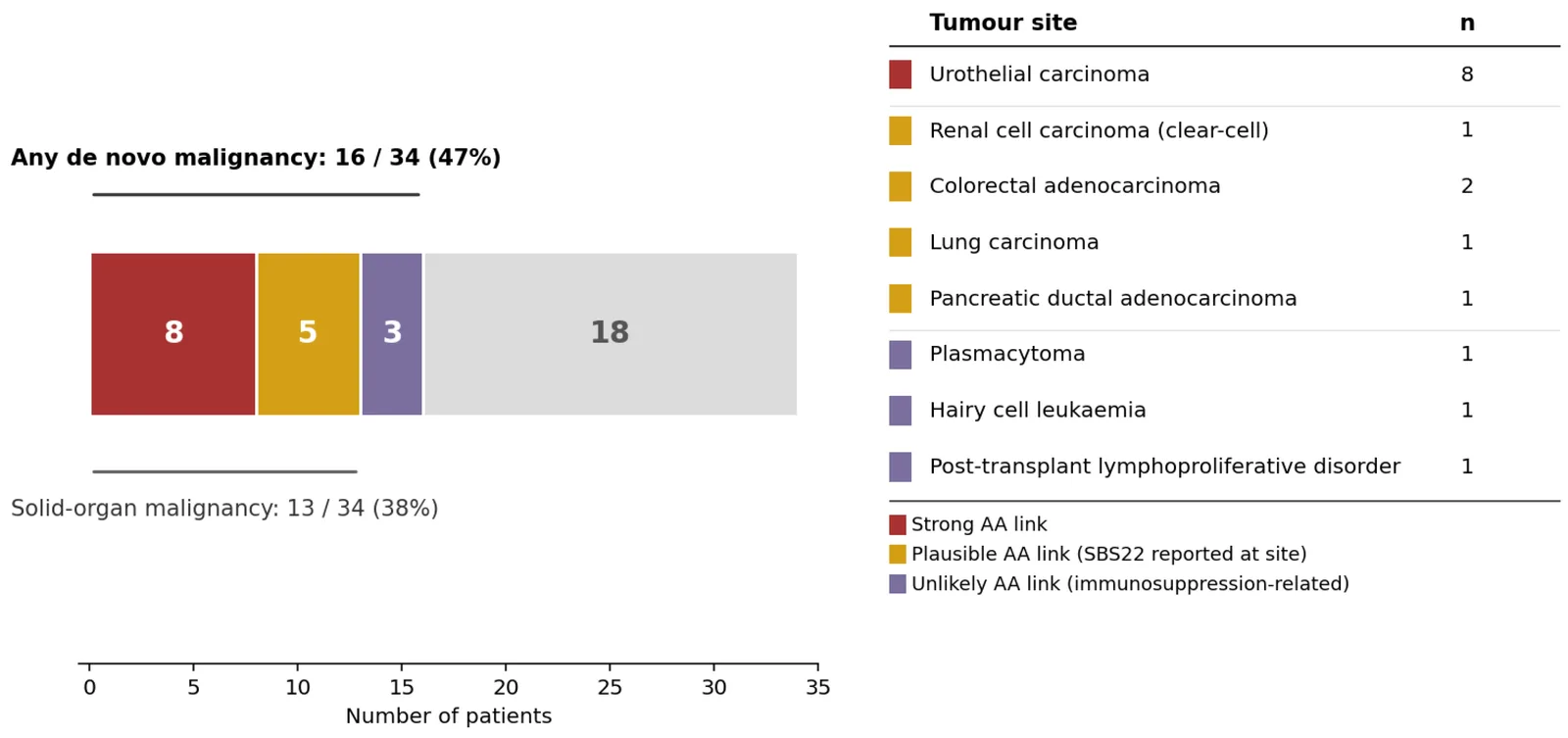
Post-transplant de novo malignant disease in the Balkan endemic nephropathy kidney-transplant cohort. Cancers are grouped by the plausibility of AA-driven carcinogenesis at the affected tumor site: strong link (upper-tract urothelial carcinoma), plausible link (sites at which the AA mutational signature SBS22 has been documented), and unlikely link (hematological malignancies attributable to long-term immunosuppression) [6,7].

Nearly half (47%) of the patients (16/34) developed some post-transplant malignant disease. Thirteen patients had a solid-organ malignant disease and three had a hematological malignancy (Figure 2). UTUC occurred in eight cases (24%) after kidney transplantation. Patient characteristics are presented in Table 1. UTUC was more frequent in females than in males; however, statistical significance was not reached. Patients who developed UTUC were five years younger at transplantation and had a shorter dialysis vintage; however, the difference was not statistically significant. New-onset diabetes after transplantation and CMV reactivation did not differ among the groups; however, Epstein–Barr virus (EBV) reactivation was significantly more common in the UTUC group. Notably, the EBV DNA status was unavailable in half of all included patients (Supplementary Table S1). The timing of the UTUC diagnosis ranged from 6 months to 15 years post-transplantation (median: 7 years).

Overall, the outcome of patients who developed UTUC was poor, with 5/8 patients dying within a year of diagnosis. Three patients underwent surgical treatment—one case with localized disease, a nephroureterectomy of the native kidney, and partial resection of the allograft had excellent outcomes and more than 10 years of cancer-free survival. In two other cases disseminated disease was discovered after surgery and outcomes were poor. Two patients died before any treatment as they presented with disseminated disease. Two patients are currently undergoing chemotherapy, and after one year of follow-up their disease remains stable. The types of treatment and outcomes are outlined in Table 2.

Eight additional patients developed malignant diseases at other sites. One patient was diagnosed with renal cell cancer at one year after transplantation; he underwent a successful nephrectomy (and later a prophylactic contralateral nephrectomy) and was cancer-free until his death from fungal pneumonia 9 years later. Four other patients had solid organ tumors (one lung adenocarcinoma, one pancreatic adenocarcinoma and two cases of colon cancer), and three had hematologic malignancies (one plasmacytoma, one post-transplant lymphoproliferative disease and one hairy cell leukemia). None of the patients with non-urothelial cancer developed UTUC.

### 3.3. Patient and Graft Survival

Five- and ten-year survival data were available for 39 patients. Seven patients died within the first five years (i.e., the five-year survival rate was 82%), whereas 22 patients (54.5%) were alive 10 years after transplantation, all with a functioning allograft. Six patients died due to malignant disease, six died from infectious causes, and one died from a cardiovascular cause, while the cause of death was unknown for six patients. Survivors and non-survivors did not differ in pre-transplant or other characteristics. Univariate logistic regression revealed no associations of dialysis vintage, age, pre-transplant diabetes, coronary artery disease, viral reactivation, or other parameters included with survival. A log-rank test did not reveal a survival benefit when comparing patients with BEN who had or had not developed UTUC at 5 years (log-rank χ^2^: 1.037, *p* = 0.308) or ten years (log-rank χ^2^ = 0.388, *p* = 0.533), most likely due to the frequently late onset of cancer.

## 4. Discussion

This study provides the longest follow-up of the large BEN kidney-transplant cohort reported to date. Malignancy risk, particularly UTUC, remained high for decades after transplantation and was associated with poor outcomes, with five of eight affected patients dying within one year of diagnosis. Five- and ten-year survival rates were 82% and 54.5%, respectively, with all 10-year survivors retaining a functioning graft.

Overall, the prevalence of UTUC after kidney transplantation in our cohort was 24%, whereas the incidence in patients without BEN is 1.35% [11]. The incidence of UTUC in chronic kidney disease (CKD) patients from endemic regions is reported to be 30–50% [12]. The incidence in our cohort was lower than that in a previous report from the Rijeka transplant center [10]. In this recently published data, the incidence of de novo UTUC in BEN kidney transplant patients was significantly higher than that in ours (58% vs. 24%) [10]. In this study, 7/12 patients developed UTUC, and it was a major cause of death as four patients died due to UTUC. Several factors probably contribute to this lower figure. First, some patients who were transplanted several decades ago could be over-diagnosed with BEN. Second, dietary exposure to AA has declined, which may translate into lower malignant potential in more recently transplanted patients. Third, incomplete surveillance data for older transplants further reduce ascertainment. Our 24% should therefore be interpreted as a lower-bound, clinically detected estimate associated with a genuinely lower biological risk. A similarly high incidence of UTUC was demonstrated in a study reporting the histopathological findings in 20 AAN patients who underwent a prophylactic nephroureterectomy—in 19 of 20 cases, cellular atypia was found, and in 55% of patients, UTUC was diagnosed. During long-term follow-up, bladder carcinoma developed in two patients (10%) [13]. A high incidence was also reported in a Belgian case series of 38 women with AAN and a transplanted kidney. Histopathology revealed that UTUC was found in 44.7% of the participants whose kidneys and ureters were removed during prophylactic nephroureterectomy. During follow-up, 39.5% of patients developed bladder urothelial carcinoma [14]. UTUC may develop years after kidney transplantation, as seen in our cohort and in the studies by Rukavina et al. and Lemy et al. [9,10,14]. In our cohort, UTUC was slightly more common in females, albeit without statistical significance. In reported studies on incidence, the male predominance typical of UTUC in unselected populations is absent and may even be reversed, particularly in the Croatian endemic areas [12,15,16]. Sex does not appear to predict oncological outcomes after radical nephroureterectomy in BEN [17]. Advanced disease at presentation was associated with a poor prognosis in our group, with more than half dying within a year of presentation. Similar results were observed in other cohorts of BEN or AAN [9,11].

Although UTUC is the malignancy most strongly associated with AA exposure, the carcinogenic effects of AA are not tissue–organ-specific. The AA mutational signature SBS22 has been detected in renal cell carcinoma from patients with CKD in endemic regions. Clear-cell RCC, hepatocellular carcinoma, intrahepatic cholangiocarcinoma, and, at low frequency, several other tumor types have been detected across East Asia [6,7,18]. In our cohort, seven patients developed non-urothelial malignancies, none of whom subsequently developed UTUC. Most of these cancers were more likely related to post-transplant immunosuppression than to AA exposure.

The screening recommendations for BEN patients at risk of developing UTUC (including transplanted patients) include biannual screening with urine cytology, ultrasound, and other screening modalities as needed [8]. Kidney transplant recipients with BEN should have a bilateral nephroureterectomy performed if they are under 65 years of age, if they are older than 65 years and have a urothelial malignancy, or if they have a positive family history of urothelial cancer. For patients who refuse prophylactic surgery, regular screening is advised. According to our data, the risk of developing UTUC does not wane over time, and screening measures should continue indefinitely. Owing to potential outcome benefits, switching patients to an mTOR inhibitor-based immunosuppressive regimen is recommended [8]. Cancer screening in this population should not be confined to the urothelium, as exposure to AA carries additional risks for the development of cancer at other sites, as discussed previously. These patients are additionally exposed to the general malignancy risk associated with long-term immunosuppression.

There are several arguments supporting prophylactic bilateral nephroureterectomy prior to kidney transplantation: 1. high UTUC incidence; 2. good prognosis for early-stage surgically curative disease; and 3. limitations of screening options. As previously discussed, UTUC is highly prevalent in kidney transplant recipients exposed to AA and represents a lifetime risk factor for UTUC (and potentially renal cell cancer) development. In real-world cohorts such as ours, the disease frequently presents at an advanced stage, which carries a dismal prognosis [9,11]. Although there are new treatment options available, immunotherapy in kidney transplant recipients means a high risk of graft rejection, further complicating the treatment. Finally, meticulous screening is also burdened with the imperfection of screening for UTUC in kidney transplant recipients—ureteroscopy of the native ureter may be technically challenging in an ESKD patient, urine cytology has low sensitivity for upper-tract disease, and cross-sectional imaging is limited by the small, contracted native kidneys typical of advanced BEN.

Taken together, these observations support bilateral nephroureterectomy at or before transplantation as the default management strategy for transplant candidates with BEN. Exceptions may be appropriate for patients older than 65 years, those with substantial surgical risk, those who decline the procedure after informed discussion, or selected individuals in whom preservation of residual native urine output outweighs the expected benefit. This position is based on the accumulated experience of endemic-region centers and international consensus recommendations for BEN/AAN transplant candidates [10], rather than randomized evidence; routine prophylactic bilateral nephroureterectomy is not universally endorsed in international urological guidelines. Therefore, management should be individualized through shared decision-making, balancing lifetime UTUC risk against surgical morbidity, loss of residual native urine output and related quality-of-life effects, age, comorbidity, and patient preference. Patients who decline surgery after informed discussion should undergo structured lifelong surveillance.

Another cautious observation in our study was the greater occurrence of EBV viremia in patients diagnosed with UTUC. Several decades ago, viral infections (including EBV) were sought after as potential causes of endemic nephropathy including UTUC; however, later studies did not support this hypothesis [1,2,3,5]. Recent studies have reported possible pathogenetic links between EBV and urothelial cancers or their invasiveness [19,20,21]. An important caveat is that EBV viremia status was missing in half of all participants, and the EBV status used was at any point within a year of the cancer diagnosis rather than at specific time points, further limiting any causality claims. It is possible that patients with UTUC were more likely to undergo more extensive testing, including EBV DNA, leading to this findings.

Accurate diagnosis and timely curative surgery are associated with favorable outcomes, whereas advanced diseases carry a poor prognosis. These findings support lifelong, meticulous surveillance. BEN should be considered in all patients with CKD who currently live in, or previously lived in, endemic regions, particularly because the disease lacks pathognomonic features and diagnosis relies on the pattern of kidney injury [8,10]. Sporadic BEN should also be excluded in patients from rural farming areas, an issue further emphasized by the recent report from the Rijeka transplant center [10,22]. Clinically, awareness must remain high because patients may originate from endemic regions but later move elsewhere [23].

The main strengths of this study are the long follow-up period and the large cohort of transplanted BEN patients. We also underlined that, if prophylactic binephrectomy was not performed, transplanted BEN patients should be closely monitored for UTUC, bladder cancer, as well as other AA-related malignancies. The main limitations include incomplete data, as reported in Supplemental Table S1. The prevalence of BEN is likely underreported due to unrecognized disease, a lack of data necessary for diagnosis and changes in diagnostic criteria. There is no doubt that several sporadic BEN cases were overlooked since this diagnosis was not accepted by many clinicians and experts in the past. Over the five-decade study period the diagnosis rested on the criteria in use at the time, which evolved from the classical epidemiological and clinical definition towards the diagnostic and classification framework subsequently formalized in the international consensus statement [8]. Nonetheless, the available data were sufficient to demonstrate the high lifetime high risk of UTUC development.

## 5. Conclusions

Kidney transplantation is a viable treatment for patients with Balkan nephropathy, but it must be accompanied by recognition of a persistent, lifelong cancer risk dominated by UTUC. The high mortality associated with advanced UTUC supports early detection and prophylactic bilateral nephroureterectomy before transplantation whenever feasible, followed by structured lifelong surveillance. The decision to proceed with prophylactic nephroureterectomy should be reached through shared decision-making by weighing the oncological benefit against surgical risk and patient preferences. Balkan nephropathy should be considered not only in patients from historically defined endemic villages but also in those from rural farming areas with compatible clinical and epidemiological features. Although the incidence and prevalence of BEN are declining because of reduced exposure to AA, new BEN-associated UTUC cases may continue to emerge because of the long latency between exposure and cancer development. Due to the lifelong cancer risk, already transplanted patients from endemic areas and those with possible exposure need indefinite, thorough surveillance.

## Figures and Tables

**Table 1:** Characteristics of the Balkan nephropathy cohort.

	Urothelial Cancer (N = 8)	No Urothelial Cancer (N = 26)	*p*
Female sex, N	5 (63%)	11 (42%)	0.43
Age at transplantation, median (IQR)	50.5 (44–62)	57.5 (52–63)	0.36
Dialysis vintage, years (SD)	2 (1–4)	5 (2–6)	0.133
Smoking, N	0	4	0.688
NODAT, N	0	4	0.365
CMV reactivation, N	2	6	0.67
EBV reactivation, N	3	2	0.009

NODAT—new-onset diabetes after transplantation; CMV—cytomegalovirus; EBV—Epstein–Barr virus.

**Table 2:** Patients with Balkan nephropathy with upper-tract urothelial cancer after kidney transplantation.

	Sex	Age	Dialysis Vintage	Time of Diagnosis (Years After Transplant)	Treatment	1 Year Cancer Survival	Outcome
1	F	65	1	7	None	No	Death before treatment
2	F	57	Unknown	Unknown	Chemotherapy	No	Death
3	F	42	3	12	Chemotherapy	Yes	Under treatment
4	F	46	2	15	Chemotherapy	Yes	Under treatment
5	M	45	2	Unknown	Surgery	Yes	Cancer-free survival
6	F	62	0.9	0.6	Surgery + Chemotherapy	No	Death
7	M	55	7	2	None	No	Death prior to treatment
8	M	41	8	1	Surgery + Chemotherapy	Yes	Death within 2 years

## Data Availability

The datasets generated and/or analyzed in the current study are not publicly available owing to patient privacy restrictions but are available from the corresponding author upon reasonable request.

## Supplementary Materials

The following supporting information can be downloaded at https://www.mdpi.com/article/10.3390/jcm15166226/s1, Table S1: Missing Data.

## Abbreviations

The following abbreviations are used in this manuscript:

UTUC	Upper-tract urothelial cancer
AA	Aristolochic acid
ESKD	End-stage kidney disease
KTR	Kidney transplant recipient
EBV	Epstein–Barr virus
CKD	Chronic kidney disease
BEN	Balkan endemic nephropathy
