# Supplementary material for: Cancer Incidence and Outcomes After Kidney Transplantation in Balkan Nephropathy Patients
Source: J Clin Med. 2026 Aug 12;15(16):6226. doi: 10.3390/jcm15166226 (PMC13513950; doi:10.3390/jcm15166226)
Supplement: Supplementary file 1 [file jcm-15-06226-s001.zip › jcm-4418801-supplementary.pdf]

Table S1. Missing Data.

Variable	Count (%)	
Age at transplantation	4	(9%)
Dialysis vintage	9	(20%)
HTN	20	(45%)
DMT2	20	(45%)
CAD	20	(45%)
Immunosuppression type	4	(9%)
CMV serostatus	18	(41%)
NODAT	19	(43%)
MACE	20	(45%)
Urothelial cancer	9	(20%)
Malignant disease	11	(25%)
Graft loss	12	(27%)
CMV reactivation	14	(32%)
EBV reactivation	22	(50%)
Survival status	5	(11%)
